# Teaching of Preventive Medicine

**Published:** 1919-05-17

**Authors:** 


### TEACHING OF PREVENTIVE MEDICINE.

A short time ago in the Guy's Hospital *Gazette*, Dr. R. King-Brown, Examiner in State Medicine to London University, gave the authorities of medical schools a clear outline of several possible and practical suggestions for the introduction of a "preventive" element into the whole system of early medical teaching. His words have been well received in many quarters, and we understand that steps have been taken in at least one of the schools of the University to follow the lines he indicates. It is pointed out that the teachings of prevention must begin with the first glimpses which the student has of the medical fields before him, and also that in the past considerations of diet, work, alcohol, various infections, and heredity have been but lightly touched upon, if at all, in any of the early text-books he encounters. It is the very beginnings of disease upon which his attention must be turned rather than the pathological states which he ultimately encounters in the wards of a hospital or the *post-mortem* room.

The Press has lately been full of explanations and praises of the new spirit of preventive medicine. The war, with its many hardships and cruelties, has at least brought realisation and demonstration of the possible achievements before us, but in the main attention has been focussed upon those men already qualified to act, the organisation of new departments to be at once complete and efficient, and the utilisation of those men whom the country has ready to hand. The coming medical generation has not been forgotten, but its requirements have been perhaps obscured by the more immediate considerations.

In the article to which we refer, the author urges that in the teaching of physiology, anatomy, pathology, etc., the teacher, himself almost invariably an experienced qualified man, should have little difficulty in carefully including every hygienic or preventive lesson which arises in connection with the subject of the moment. Even in surgery and clinical medicine, though the immediate aspects of the case may be pathological, every attention may be given to a review of the prevention or prophylaxis of the condition demonstrated.

Finally, the attitude of examiners is considered. If examining bodies lay more stress upon the importance of the earliest signs of disease, counter-measures and the like, the student must inevitably make himself more fully acquainted with these aspects of his future professional duties.

These are but a few of the points to which Dr. King-Brown would call our attention, and once set upon this line of thought we leave the reader to picture for himself how greatly such a revision of teaching would tend to help preventive medicine of the future.

